# Alcohol-related emergency department visits and income inequality in New York City, USA: an ecological study

**DOI:** 10.4178/epih.e2019041

**Published:** 2019-10-08

**Authors:** Kathleen H. Reilly, Katherine Bartley, Denise Paone, Ellenie Tuazon

**Affiliations:** 1 Bureau of Epidemiology Services, New York City Department of Health and Mental Hygiene, New York, NY, USA; 2 Bureau of Alcohol and Drug Use Prevention, Care and Treatment, New York City Department of Health and Mental Hygiene, New York, NY, USA

**Keywords:** Socioeconomic factors, Alcohol drinking, Urban health, New York City

## Abstract

**OBJECTIVES:**

Previous research has found that greater income inequality is related to problematic alcohol use across a variety of geographical areas in the USA and New York City (NYC). Those studies used self-reported data to assess alcohol use. This study examined the relationship between within-neighborhood income inequality and alcohol-related emergency department (ED) visits.

**METHODS:**

The study outcome was the alcohol-related ED visit rate per 10,000 persons between 2010 and 2014, using data obtained from the New York Statewide Planning and Research Cooperative System. The main predictor of interest was income inequality, measured using the Gini coefficient from the American Community Survey (2010-2014) at the public use microdata area (PUMA) level (n=55) in NYC. Variables associated with alcohol-related ED visits in bivariate analyses were considered for inclusion in a multivariable model.

**RESULTS:**

There were 420,568 alcohol-related ED visits associated with a valid NYC address between 2010 and 2014. The overall annualized NYC alcohol-related ED visit rate was 100.7 visits per 10,000 persons. The median alcohol ED visit rate for NYC PUMAs was 88.0 visits per 10,000 persons (interquartile range [IQR], 64.5 to 133.5), and the median Gini coefficient was 0.48 (IQR, 0.45 to 0.51). In the multivariable model, a higher neighborhood Gini coefficient, a lower median age, and a lower percentage of male residents were independently associated with the alcohol-related ED visit rate.

**CONCLUSIONS:**

This study found that higher neighborhood income inequality was associated with higher neighborhood alcohol-related ED visit rates. The precise mechanism of this relationship is not understood, and further investigation is warranted to determine temporality and to assess whether the results are generalizable to other locales.

## INTRODUCTION

Poverty is associated with poor health outcomes, including diabetes, coronary heart disease, and mortality [1]. This association is thought to be mediated through material deprivation, including lack of health resources, which can manifest as limited access to health care services, difficulty paying for food and housing, and differences in the quality of the social and physical environment [2]. Although the relationship between poverty and health has been well studied, the relationship between income inequality and health is less thoroughly understood. Studies have found that income inequality is associated with greater mortality, lower self-rated health, and other poor health outcomes [3-5]. Over the past 4 decades, income inequality has increased in the USA and New York City (NYC) [6,7]. Income inequality has been postulated to increase stress, which may result in an increased frequency of stress-reducing behaviors, including alcohol and drug use [3]. Furthermore, health outcomes related to neighborhood economic status may vary by race and ethnicity [8,9].

The relationship between poverty and substance use is nuanced and is mitigated by environmental and individual factors [10]. Along similar lines, the association between neighborhood poverty and problematic alcohol use is not well understood, and researchers have found both positive and null results [11,12]. Previous research has found that income inequality is related to problematic alcohol use in the USA [13] and NYC [14]. Problematic alcohol use has been defined in a variety of ways, including by the frequency and quantity of alcohol consumed, the frequency of drinking to intoxication, and the rates of alcohol-attributable hospitalizations and deaths [14-16]. Excessive alcohol consumption is associated with premature death, cancer, liver cirrhosis, fetal alcohol syndrome, injury, crime, and increased healthcare costs [17]. In NYC between 2010 and 2014, there were 1,082 deaths due to alcohol use [18]. In the USA, the number of alcohol-related emergency department (ED) visits increased sharply between 2006 and 2014; these visits may be a harbinger of other deleterious health outcomes [19,20].

Understanding the relationship between income inequality and problematic alcohol use could help inform neighborhood-level interventions to prevent adverse outcomes of alcohol use. NYC is an ideal place to study income inequality, given that the metropolitan area has one of the highest levels of income inequality in the USA [21]. The current study examines the relationship between alcohol-related ED visits and income inequality at the neighborhood level in NYC.

## MATERIALS AND METHODS

### Neighborhood definition

Neighborhoods were defined as public use microdata areas (PUMAs), which were developed by the US Census Bureau and have populations of at least 100,000 people. There are 55 PUMAs in NYC, with a median population size of approximately 145,000. NYC community districts (CDs) are based on PUMAs. While there are 55 PUMAs in NYC, there are 59 CDs. Each CD has a community board, which is the lowest level of city government, providing an advisory role in land use, budgeting, and the delivery of city services to the community. CD boundaries were developed by the NYC government based on historic and geographic communities without respect to US Census geographical units. CDs have previously been used to represent neighborhoods in other studies [14,22]; however, because census data are available at the PUMA level, the PUMA was the geographic unit chosen to represent neighborhoods in the current study.

### Measures

Three variables were used to assess neighborhood inequality. The Gini coefficient is a commonly used income inequality measure ranging from 0 to 1, with values closer to 0 representing less inequality and those closer to 1 representing more inequality. Gini coefficient data based on household income were downloaded directly from the 2010-2014 American Community Survey (ACS) at the PUMA level. To incorporate race into the measure of inequality, poverty ratios were developed similar to those used by Karriker-Jaffe et al. [13]; these were calculated for each PUMA by dividing the percentages of those with income below the poverty level in the past 12 months as follows: (1) the percentage for Blacks divided by that for Whites and (2) the percentage for Hispanics divided by that for Whites.

ED visit information was derived from the New York Statewide Planning and Research Cooperative System (SPARCS). Alcohol-related ED visits were tabulated for people between the ages of 13 and 84 with an International Classification of Diseases, Ninth Revision (ICD-9) code for the principal diagnosis or any other diagnosis (excluding admitting diagnosis) of a chronic condition 100% attributable to alcohol (alcohol psychosis [ICD-9 code 291], alcohol abuse [303.0, 305.0], alcohol dependence syndrome [303.9], alcohol polyneuropathy [357.5], alcohol cardiomyopathy [425.5], alcoholic gastritis [535.3], or alcoholic liver disease [571.0-571.3]) or of an acute condition 100% attributable to alcohol (excessive blood level of alcohol [ICD-9 code 790.3] or alcohol poisoning [980.0, 980.1, E860.0, E860.1, E860.2, E860.9]) [23]. Only cases of patients who were treated in and discharged from the ED (not transferred to other departments) were included in the analysis. Patients’ residential address data were geocoded to the PUMA level, alcohol-related ED visit rates were determined using total PUMA populations according to the ACS (2010-2014), and rate data were annualized by dividing by 5.

Demographic variables were taken from the ACS (2010-2014) data. These variables included percent race/ethnicity (non-Hispanic White, non-Hispanic Black, non-Hispanic Asian, non-Hispanic other, or Hispanic), median age, percent male, median personal income, percent living in poverty in the past 12 months, percent unemployed of the civilian labor force (which included individuals 16-64 years of age), and percent of persons ≥25 years of age with less than a high school education. Data describing the density of alcohol and wine retailers and bars were derived from the State Liquor Authority Brand Label and Wholesaler Information for Alcoholic Beverage Products Registered in New York State. Although a variety of establishments are licensed to sell alcohol in New York State, the current study included only retailers that primarily sell alcohol for personal consumption (liquor stores, wine stores, and bars).

### Statistical analyses

The relationship between the PUMA alcohol-related ED visit rate and the Gini coefficient was explored using a scatter plot and the calculation of R^2^. Medians and interquartile ranges were calculated for each variable. Variables with a theoretical basis that were found to be significantly associated with alcohol-related ED visits in the bivariate analysis were considered for inclusion in the multivariable model. Linear regression models were weighted using the inverse of the population of each PUMA. Variables were excluded from the multivariable model using backwards selection for variables with p-value>0.1, after which collinearity was assessed and variables with a variance inflation factor (VIF) ≥5 were also excluded from the model.

An ecological study design was chosen over a multilevel analysis given the aim to examine contextual neighborhood effects, as well as the paucity of individual-level data available. In order to provide limited information about individuals with alcohol-related ED visits, an ancillary analysis was performed to compare individual sex, age, and Medicaid payment for any portion of the ED visit (used as a proxy for income) for those with alcohol-related ED visits from the PUMAs in the top quartile (representing the largest degree of income inequality) with those living in PUMAs in the bottom quartile (representing the smallest degree of income inequality). Significant differences for individual characteristics were identified using the Wilcoxon signed-rank test for non-normal continuous data and the chi-square test for categorical data. Analyses were conducted using R version 3.4.3 (https://cran.r-project.org/bin/windows/base/old/3.4.3/: stats, sp, rgdal, and nortest).

### Ethics statement

The study protocol was approved by the Institutional Review Board of the New York City Department of Health and Mental Hygiene (IRB No. 16-123) as research involving materials that were collected for non-research purposes.

## RESULTS

**Figure 1.**
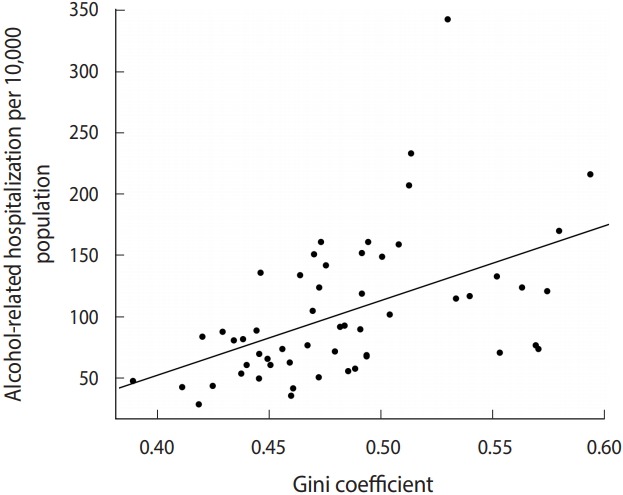
Alcohol-related hospitalizations (2010-2014) and Gini coefficients for New York City public use microdata area (R^2^ =0.25).

Between 2010 and 2014, there were 419,807 alcohol-related ED visits for persons aged 13 to 84 associated with a valid NYC address. The overall annualized NYC alcohol-related ED visit rate was 100.5 visits per 10,000 persons. Table 1 shows the median and interquartile range (IQR) values for the alcohol-related ED visit rate, Gini coefficient, racial/ethnic poverty ratios, bar density, liquor and wine store density, and percentages of demographic characteristics at the PUMA level. The median alcohol-related ED visit rate for NYC PUMAs was 88.0 visits per 10,000 persons (IQR, 64.5 to 133.5), and the median Gini coefficient was 0.48 (IQR, 0.45 to 0.51). Figure 1 shows the PUMA Gini coefficients plotted against the alcohol-related ED visit rates. The R^2^ value for the relationship between alcohol-related ED visit rate and Gini coefficient was 0.25 (Figure 1).

Table 2 shows the relationship between neighborhood factors and alcohol-related ED visit rates. A higher Gini coefficient, higher liquor and wine store density, higher percentage of people living in poverty in the past 12 months, higher percentage of people unemployed (of individuals ages 16-64), lower median age, lower percentage of males, lower percentage of Asians, and lower percentage of foreign-born individuals were each associated with the alcohol-related ED visit rate in the bivariate analysis. In the multivariable model, a higher Gini coefficient, lower median age, and lower percentage of male individuals were independently associated with alcohol-related ED visit rates. The VIFs for all variables in the final multivariate model were less than 5.

A comparison of persons with alcohol-related ED visits from neighborhoods with Gini coefficients in the bottom quartile (least unequal, Gini≤0.448) with those in the top quartile (most unequal, Gini≥0.506) found that those in the most unequal PUMAs tended to be older (median, 48 years; IQR, 36 to 55) than those in the least unequal PUMAs (median, 43 years; IQR, 30 to 52, Wilcoxon p<0.001). A larger percentage of alcohol-related ED visits were paid for using Medicaid by persons from the most unequal PUMAs than by those from the least unequal PUMAs (least unequal PUMAs: 39.7% vs. most unequal PUMAs: 56.9%; χ^2^=6062.3, p<0.001). The percentage of alcohol-related visits for men was slightly higher in the least unequal PUMAs (78.7%) than in the most unequal PUMAs (77.6%) (χ^2^=34.8, p<0.001).

## DISCUSSION

To our knowledge, this is the first study to examine and find an association between neighborhood income inequality, as measured by the Gini coefficient, and alcohol-related ED visits. The results of this study are consistent with the findings of other research studies, which have explored the relationship between income inequality and problematic alcohol use [13-16]. However, Henderson et al. [24] did not find income inequality to be associated with alcohol dependence at the state level. Although the Gini coefficient was significantly associated with the rate of alcohol-related ED visits, neither of the racial/ethnic poverty ratios were significantly associated with alcohol-related ED visits, which conflicts with the findings of the nationally representative study conducted by Karriker-Jaffe et al. [13] that explored the relationship between inequality and problematic alcohol use at the state level. The lack of an association between racial/ethnic poverty ratios and alcohol-related ED visits may be explained by the fact that NYC is segregated, particularly with respect to Black populations [25]. The choice of geographical units used in this analysis might have contributed to this null finding, as smaller geographical units are likely to be more segregated than the five boroughs or the city overall. Lower variability at the smaller geographic level may have tempered the relationship between racial/ethnic poverty ratios and alcohol-related ED visits. Neighborhood median personal income was also not associated with rate of alcohol-related ED visits. Galea et al. [14] found greater alcohol use in neighborhoods with higher median personal income; however, that study did not examine alcohol use resulting in ED visits.

The current study highlights that neighborhood factors may be important determinants of health, specifically with regard to alcohol-related ED visits. It is unknown whether a threshold exists at which income inequality impacts health outcomes, and little is also known about how that effect may vary for different health outcomes [26]. Kawachi et al. [27] hypothesized that in some circumstances, the neighborhood context may supersede individual resources, and disinvestment in social capital may mediate the relationship between income inequality and increased mortality. Tumin [28] asserted that social hierarchy inhibits social integration through hostility, suspicion, and distrust. Additional research is warranted to further explore the relationship between income inequality and alcohol-related ED visits, as well as potential mediators of this relationship.

In addition to neighborhood income inequality, neighborhoods with younger populations and larger percentages of females exhibited higher alcohol-related ED visit rates. Older adults tend to disapprove of heavy alcohol use and be more aware of its potential harms, and younger neighborhoods may have more permissive drinking norms [29]. The relationship between neighborhood percentage of male individuals and alcohol-related ED visit rate is less clear, especially considering that among alcohol-related ED visits, the percentage of male patients is around 80%, as indicated by the results of the current study’s ancillary analysis. Male individuals are more likely to engage in problematic alcohol use [30]; however, in the current study, the greater the percentage of male individuals in a neighborhood, the lower the alcohol-related ED visit rate. The association between bar density and alcohol-related ED visits was not significant, perhaps because the neighborhood of residence may be different than the neighborhood where alcohol consumption takes place. However, the association between liquor and wine store density and alcohol-related ED visits was significant at the bivariate level; alcohol outlet density has previously been found to be associated with increased alcohol consumption and harm [31].

The ancillary analysis in the current study revealed that the neighborhoods with the highest income inequality had a greater percentage of alcohol-related ED visits paid for with Medicaid than the neighborhoods in the lowest quartile of income inequality. This finding indicates that those with lower income living in neighborhoods with high income inequality may be at the greatest risk for alcohol-related ED visits, although further research is warranted to further explore this relationship and determine the mechanism influencing this association. Analogously, a study conducted in South Korea found that among those with alcohol-related problems, those with lower incomes displayed a higher mortality rate than those with higher incomes [32]. The ancillary analysis also found that among those with history of an alcohol-related ED visit, those living in neighborhoods with the highest quartile of income inequality were older than those living in neighborhoods with the lowest quartile of income inequality. Older people and younger people may be affected differentially by neighborhood inequality, and long-term residents could experience stress if they live in gentrifying neighborhoods [33]. Although the proportion of male individuals among alcohol-related ED visits was statistically significantly lower for those living in the highest quartile of income inequality compared with those living in the lowest quartile of income inequality, it is doubtful that this difference is practically significant and is instead likely due to the large sample size [34].

Chapple [35] stated that US cities are currently in the midst of an income crisis, with real wages below what they were in the 1970s, resulting in increased inequality and facilitating gentrification. Kennedy et al. [36] suggested that even a modest redistribution of wealth could have an impact on community health. NYC and New York State have implemented several policies that have the potential to reduce income inequality, including mandating paid family leave, legislating tuition-free college, providing affordable housing, decreasing the sex wage gap, increasing the minimum wage, and supporting economic democracy through union representation. Place-based interventions should also be considered to reduce the potential negative health impacts associated with income inequality [37].

### Limitations

This study was subject to several limitations. This study analyzed aggregate data rather than individual data; however, an “unmixed” ecological study, as opposed to the use of multilevel models, is appropriate given that the analysis aimed to examine contextual neighborhood effects, and the main exposure of interest (income inequality) can only be measured at a group level [27,38]. However, inferences at the ecological level should not be extended to the individual level. It is possible that there were other factors, both concurrent and historical, that were not accounted for in this analysis; these unmeasured variables may confound the relationship between income inequality and alcohol-related ED visits, and the ecological nature of this study presents additional challenges in the attempt to control for confounding variables [39]. The data in the current study were not deduplicated by individual patient, since the ED visit data contained limited identifiers; therefore, it is possible that in some neighborhoods, certain individuals disproportionately visit the ED for alcohol-related conditions, thereby inflating the neighborhood ED visit rate. Given the structure of SPARCS data, hospitalization data were limited to treat-and-release ED visits; inclusion of visits that required transfer to an inpatient facility and visits to urgent care facilities may have resulted in different findings. Furthermore, this study considered alcohol-related ED visits for the principal diagnosis and any other diagnoses, but it did not address comorbidities for these visits nor diagnoses that were not 100% attributable to alcohol, which could differentially impact neighborhood populations. To the authors’ knowledge, the validity of alcohol-related diagnosis codes in New York State ED data has not been assessed, and it is possible that the current study may have underestimated the number of alcohol-related hospitalizations if alcohol use was not recorded in patients’ records. Accordingly, the results of this study could be biased if alcohol-related ED visits were differentially underreported by the PUMA of residence. The cross-sectional nature of this study makes it difficult to draw conclusions about temporality, given that income inequality may have a lag effect up to 15 years [26]. The alcohol retailers included in the density measures were not exhaustive, as grocery stores and drug stores that may sell beer, micro-breweries and distilleries that also function as bars, and restaurants that serve alcohol were not included. Research is inconclusive as to how grocery store alcohol sales compare with alcohol-only outlets, and it is unknown how retailers that do not primarily sell alcohol may impact alcohol-related ED visits [40]. The data used to create the bar and liquor and wine store density measures were taken at the time the data were downloaded (March 12, 2014) and may not be reflective of businesses open at any point during the entire study period (2010-2014), however, we do not expect that the locations of alcohol retailers changed much over this time frame. Furthermore, this analysis was based on NYC data and may not be generalizable to other jurisdictions. Income inequality in NYC may be different than other urban areas or other regions, especially considering that income inequality does not consider usage of social welfare services, the availability of which varies by region. Results demonstrating a relationship between income inequality and problematic alcohol use in regions such as the USA, Europe, and Australia indicate that the association between alcohol-related ED visits and income inequality likely persists outside of NYC [13,15,16]. This study found a significant relationship between alcohol-related ED visits and income inequality at the PUMA level; however, studies with other scales may come to different conclusions [26].

In conclusion, income inequality was associated with alcohol-related ED visits, a manifestation of unhealthy drinking, at the neighborhood level. Efforts to reduce income inequality may have an impact on reducing the rates of alcohol-related ED visits. Future research is needed to better understand the mechanism of how income inequality relates to alcohol-related ED visits. Future studies should examine the impact of policies to reduce income inequality, whether these policies have an effect on problematic alcohol use, and whether effects vary across jurisdictions.

## Figures and Tables

**Table 1.** Median and range of included NYC PUMA study variables^1^

Variables	Median (interquartile range)
Alcohol hospitalization rate (per 10,000 population)	88.0 (64.5, 133.5)
Gini coefficient	0.48 (0.45, 0.51)
Black:White poverty ratio	1.5 (1.0, 2.3)
Hispanic:White poverty ratio	1.8 (1.3, 2.3)
Bar density (per 10,000 population)	5.0 (2.4, 7.4)
Liquor and wine store density (per 10,000 population)	1.5 (1.2, 1.9)
Unemployed (of individuals ages 16-64), %	10.3 (8.0, 13.8)
Less than high school graduate (of individu- als ages 25+), %	19.7 (13.9, 26.8)
Median age, yr	35.3 (33.7, 38.4)
Male, %	47.3 (46.3, 49.0)
Hispanic, %	22.3 (14.0, 41.6)
White, %	28.0 (11.9, 53.9)
Black, %	13.1 (3.7, 30.8)
Asian, %	8.0 (3.5, 15.5)
Other race, %	2.2 (1.7, 2.8)
Foreign-born, %	36.0 (26.9, 45.4)
Living in poverty in the past 12 mo, %	19.8 (13.9, 28.1)
Median personal income, US$	25,904 (21,964, 31,716)

NYC, New York City; PUMA, public use microdata area.

1 All variables are continuous.

**Table 2.** Associations with PUMA alcohol-related emergency department hospitalization rate using linear regression models weighted by the inverse of the population of each PUMA

Variables	Bivariate coefficient (SE)	Multivariable coefficient (SE)
Gini coefficient	657.8 (147.4)^**^	479.6 (130.9)^**^
Black:White poverty ratio	1.7 (7.6)	
Hispanic:White poverty ratio	-9.1 (10.7)	
Bar density (per 10,000 population)	0.7 (0.5)	
Liquor and wine store density (per 10,000 population)	22.4 (11.3)^*^	
Unemployed (of individuals ages 16-64), %	4.5 (2.2)^*^	
Less than high school graduate (of individuals ages 25+), %	1.5 (0.8)	
Median age, yr	-7.5 (1.7)^**^	-6.5 (1.5)^**^
Male, %	-8.3 (3.9)^*^	-6.8 (3.0)^*^
Hispanic, %	0.6 (0.4)	
White, %	-0.6 (0.3)	
Black, %	0.6 (0.3)	
Asian, %	-1.6 (0.7)^*^	
Other race, %	-2.2 (3.2)	
Foreign-born, %	-1.4 (0.6)^*^	
Living in poverty in past 12 mo, %	3.1 (0.7)^**^	
Median personal income, US$	-2.2×10-4 (5.9×10-4)	

PUMA, public use microdata area; SE, standard error.

* p<0.05,

** p<0.01.
